# Determinants of retinopathy and short-term neurological outcomes after cerebral malaria

**DOI:** 10.1038/s41598-025-97468-4

**Published:** 2025-04-19

**Authors:** Florence Bodeau-Livinec, Agnès Aubouy, Inès Besnard, Karl Angendu, Josselin Brisset, Jade Royo, Elisée Kinkpe, Linda Ayedadjou, Audrey Mowendabeka, Thomas Lathière, Farid Boumediène, Ida Dossou-Dagba, Jules Alao, Jean-François Faucher

**Affiliations:** 1 Ecole des hautes études en santé (EHESP), Institut de recherche en santé, environnement et travail (IRSET), 93210 Saint-Denis, France; 2 UMR152 PHARMADEV, Research Institute for Development (IRD), Toulouse 3 University, Toulouse, France; 3 Inserm U1094, IRD U270, Univ. Limoges, CHU Limoges, EpiMaCT - Epidemiology of chronic diseases in tropical zone, Institute of Epidemiology and Tropical Neurology, OmegaHealth, Limoges, France; 4 National Public Health Institute (INSP), Kinshasa, Democratic Republic of Congo; 5 Infectious Diseases and Tropical Medicine Department, University Hospital, 2 Avenue Martin Luther King, 87000 Limoges, France; 6 Paediatric Department, Reference Hospital Centre, Abomey Calavi, Benin; 7 Paediatric Department, Mother and Child University and Hospital Center (CHU-MEL), Cotonou, Benin; 8 Department of Neonatal Intensive Care Unit, University Hospital, Limoges, France; 9 INSPEARS Limoges Simulation Center, Limoges Medical School, Limoges, France; 10 Ophtalmology department, University Hospital, Limoges, France

**Keywords:** Cerebral malaria, Neurological deficit, Retinopathy, Children, West Africa, Immune markers, Cytokines, Neurological manifestations, Malaria, Paediatric research

## Abstract

Neurological abnormalities are frequent after cerebral malaria (CM) resolves. The identification of survivors that should be prioritized during follow-up after CM is necessary for post-hospitalization care. We analysed social, clinical, and immune determinants of malarial retinopathy (MR) and short-term neurological outcomes after CM. Children aged 24 to 71 months with CM were prospectively followed-up until 28 days after admission at two hospitals in Benin. Direct ophthalmoscopy was performed shortly after admission. Plasma biomarkers were measured at admission. A neurocognitive deficit screener was administered at discharge and 21–28 days after admission. Of 70 children, 20 died before discharge (28.6%). Neurological deficits decreased from 100% on admission to 48.9% at discharge, and to 16.7% at 21–28 days after admission. MR was found in 58% of children. In multivariate analysis, factors associated with MR were a traditional consultation before admission and study site. In addition, neurological deficits were associated with MR (Odds Ratio 5.54 95% CI (1.30–23.54)). In univariate analysis, higher plasma levels of angiopoietin-2 were associated with neurological deficit at discharge and at days 21–28 post-admission. Therefore, MR and endothelium activation may be markers of neurological deficit, the former at hospital discharge and the latter at discharge and at D21–D28 post-admission.

**Supplementary Information:**

The online version contains supplementary material available at 10.1038/s41598-025-97468-4.

## Background

Since 2015, the progress observed so far in terms of malaria prevalence and mortality with implementation of the World Health Organization Roll Back Malaria program has stalled^1^. Cerebral malaria (CM) is by far the most common cause of non-traumatic coma in Benin, West Africa, and has been associated with a poor prognosis even during the artesunate era^2^. Although malaria eradication within a generation is the goal, the number of deaths related to malaria remains high, especially in areas of West and Central Africa^3^.

Neurological sequelae are also observed after CM in paediatric survivors and its incidence in African children varies widely among studies, from 6 to 31% of cases^4,5^. Neurocognitive impairments include motor, speech, language, and memory domains^6,7^. Seizures may also occur months after a CM episode^5^.

All children with CM should therefore be followed-up for several months after discharge^4,8^ but it is logistically difficult to do so in areas with few resources. Some studies have reported decreases in the proportion of children with CM-related gross deficits 3–8 weeks after discharge^5,9^. As the epidemiology of severe malaria may shift^10^, the extent to which disabilities change within weeks after discharge needs to be reassessed. Furthermore, predicting which children admitted with CM would be most likely to have short-term, post-discharge disabilities would be helpful to prioritize such individuals for follow-up.

Although malarial retinopathy (MR) may improve the specificity of CM diagnosis^11,12^ and predict the risk of sequelae in children with CM^8^, it is rarely performed in routine practice in malaria-endemic areas, even in tertiary care hospitals. MR is associated with reduced perfusion of the retina and may mirror reduced brain perfusion possibly related to sequestration of parasitised erythrocytes^12,13^. Modifications of the brain blood barrier may explain retinal haemorrhage^12^. MR is also related to markedly increased brain volume as evidenced by MRI imaging^14^. Few studies have reported an association between MR and increased mortality^15^, and even fewer have explored MR as a predictor of neurological outcomes in survivors^8,16^.

Biological markers of clinical outcomes including neurological deficits would be useful for identifying children most at risk of developing neurological deficits. Few studies have focused on biological prognostic markers for neurologic sequelae following CM. However, inflammatory and oxidative responses are associated with mortality and severity during malaria^17–20^. Cytokines, including chemokines, are highly relevant biomarkers of inflammation, displaying pro- or anti-inflammatory properties, recruiting and activating immune cells, and thus modulating their microenvironment^21^. Leukotrienes (LTB4, LTC4, LTD4, LTE4), lipoxins (LXA4, LXB4), prostaglandins (PGD2, PGE2, PGF2, PGI2), and all products of the arachidonic acid metabolism pathway are also involved in pro- and anti-inflammatory equilibrium^22–24^. LXA4, an anti-inflammatory mediator that inhibits LTB4 and thus neutrophil function and migration, limits endothelial dysfunction and prevents breakdown of the blood brain barrier when administered to infected mice^25^. Regarding the oxidant response, isoprostane and glutathione (in its reduced and oxidized forms, GSH and GSSG, respectively) are involved in the arachidonic acid metabolism pathway and associated with disease resolution in CM^19,20^. In this work, we studied the plasma levels of pro- and anti-inflammatory cytokines, along with urine biomarkers of the inflammatory and oxidative response, in relation to MR in children with CM and to neurocognitive deficits at discharge and 21–28 days post-admission.

This work is part of a larger project called NeuroCM^26^, whose main goal was to identify the causative and remedial factors of neuroinflammation in the context of CM. In this study, we studied the determinants of MR observed early after admission and neurological deficits observed at discharge and 21–28 days post-discharge.

## Results

### Study population

**Fig. 1:**
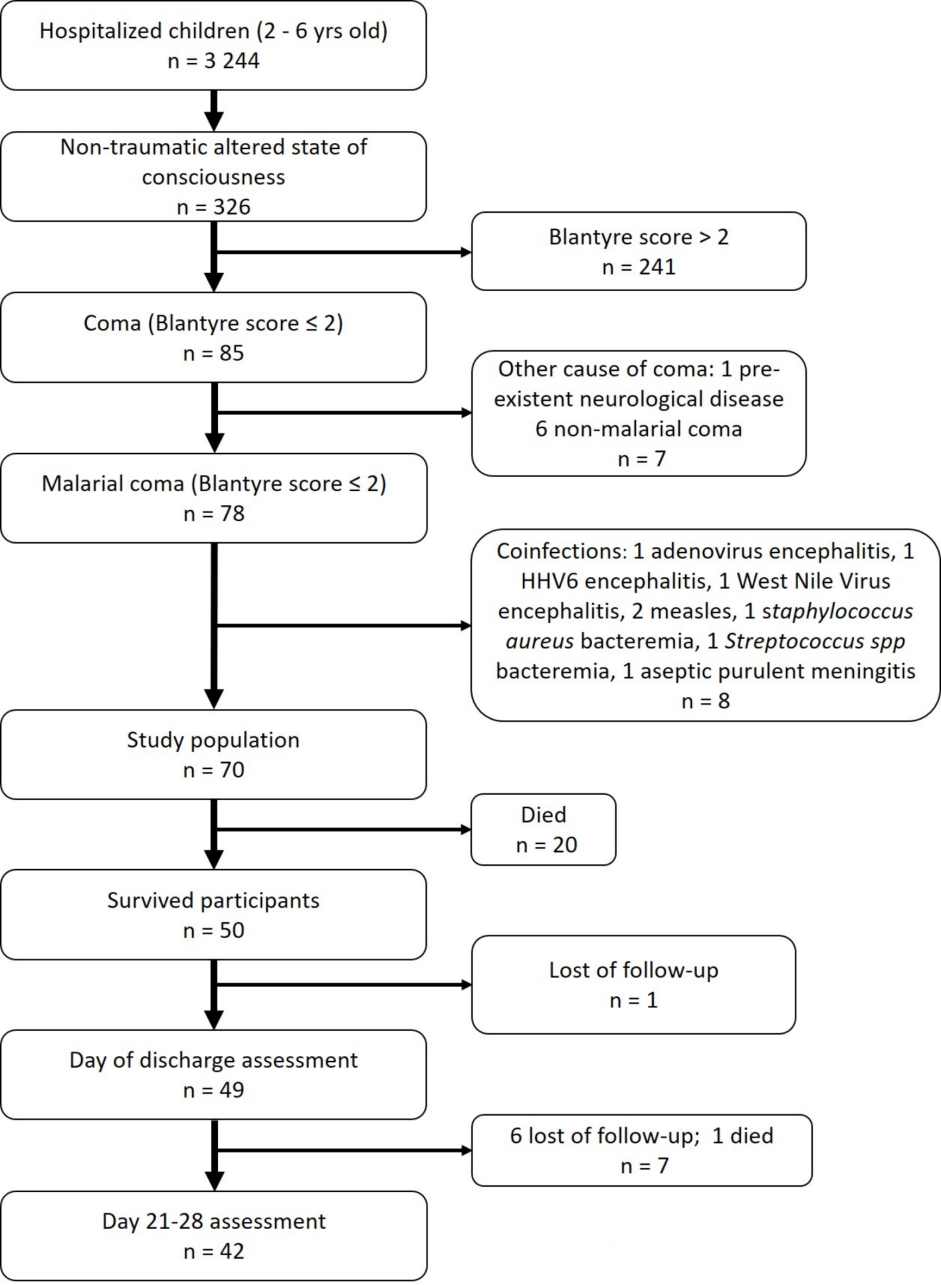
Flow diagram of the study participants.

From March 1 to November 30, 2018, 326 children aged between 2 and 6 years were admitted with coma to the hospital (Fig. 1). Of these, one child had pre-existent neurological disease, six were in a non-malarial coma, and a further eight had a coinfection, all of which were excluded. Overall, 70 children met the inclusion criteria; 38 were at Calavi Hospital and 32 were at CHUMEL. Female/male repartition was 41/29, with a male–female sex ratio of 0.7. The median age was 43.3 (36.1–54.1) months. The median duration between the start of symptoms and admission at the hospital was 4 days (SD = 1.84). Among survivors at discharge, the median duration between inclusion in the study and discharge from the hospital was 6.9 days (SD = 3.1). The case fatality rate was 28.6% (20/70). Study population characteristics according to vital status at discharge are provided in Table 1. A Blantyre coma score below 2, jaundice, hypoglycaemia, and acidosis were associated with a fatal outcome in univariate analyses.

**Table 1:** Study population characteristics.

	Deceased (*n* = 20) *n* (%)	Non-deceased (*n* = 50) *n* (%)	*p*-value
Age			0.389
4 years old or less	11 (55.0)	33 (66.0)	
More than 4 years old	9 (45.0)	17 (34.0)	
Gender			0.357
Female	10 (50.0)	31 (62.0)	
Male	10 (50.0)	19 (38.0)	
Admitting hospital			0.255
Calavi	13 (65.0)	25 (50)	
CHUMEL	7 (35.0)	25 (50)	
Medical consultation			1.000
Yes	18 (90.0)	45 (90.0)	
No	2 (10.0)	5 (10.0)	
Traditional consultation			0.819
Yes	9 (45.0)	21 (42.0)	
No	11 (55.0)	29 (58.0)	
*Pre-admission treatment*			
Antibiotic			**0.105**
Yes	9 (45.0)	33 (66.0)	
No	11 (55.0)	17 (34.0)	
Anti-epileptic			**0.053**
Yes	4 (20.0)	22 (44.9)	
No	16 (80.0)	27 (55.1)	
Onset of illness with coma			0.213
Yes	16 (80.0)	46 (92.0)	
No	4 (20.0)	4 (8.0)	
*Clinical and biological examinations*			
Blantyre’s score			0.007*
2	9 (45.0)	39 (78.0)	
0 or 1	11 (55.0)	11 (22.0)	
Multiple convulsions			0.939
Yes	10 (50.0)	24 (49.0)	
No	10 (50.0)	25 (51.0)	
Respiratory distress			**0.068**
Yes	8 (40.0)	9 (18.0)	
No	12 (60.0)	41 (82.0)	
Jaundice or elevated bilirubin > 50 µmol/L			0.004*
Yes	15 (75.0)	18 (36.7)	
No	5 (25.0)	31 (63.3)	
Hypoglycaemia (glucose < 2.2 mmol/L)			< 0.001*
Yes	14 (70.0)	11 (22.0)	
No	6 (30.0)	39 (78.0)	
Acidosis (plasma bicarbonate < 15 mmol/L)			< 0.001*
Yes	17 (85.0)	16 (32.6)	
No	3 (15.0)	33 (67.4)	
Severe anaemia (haemoglobin < 50 g/L, haematocrit < 15%)			1.000
Yes	8 (40.0)	20 (40.0)	
No	12 (60.0)	30 (60.0)	

p-value < 0.20 are in bold. *p-value < 0.05.

### Assessment of neurological status

Of the 50 children who survived CM, 49 had a neurological assessment (withdrawal of one child happened on the day before scheduled discharge). Neurological assessment at discharge (Table 2) was carried out in 49 children and 7 children were lost to follow-up by D21–28. Findings describing neurological assessments are detailed in Table 2. In the 42 children with questionnaires available both at discharge and at D21–28, neurological deficits were found more frequently at discharge than at D21–28 post-admission (48.9% vs. 16.7%, *p* < 0.001).

**Table 2:** NeuroCognitive deficits screener at day of discharge and D21–D28 post-hospitalization.

	Day of discharge (*N* = 49) *n*/*N* (%)	D21–D28 post-hospitalization (*N* = 42) *n*/*N* (%)
NeuroCognitive deficits screener		
Does the child have any serious delay in sitting?	6/49 (12.2%)	2/42 (4.8%)
Does the child have any serious delay in standing?	17/49 (34.7%)	5/42 (11.9%)
Does the child have any serious delay in walking?	20/49 (40.8%)	4/42 (9.5%)
Does the child have difficulty seeing either in the day time or at night?	2/49 (4.1%)	2/42 (4.8%)
Does the child appear to have difficulty hearing?	1/49 (2.0%)	2/42 (4.8%)
When you tell the child to do something, does he/she seem to understand what you are saying?	47/49 (95.9%)	40/42 (95.2%)
Does the child speak at all (can he or she make himself or herself understood in words; can he or she say any recognizable words)?	40/49 (81.6%)	39/42 (92.9%)
Does the child appear in any way mentally backward, dull, or slow?	6/47 (12.8%)	4/42 (9.5%)
Does the child have difficulty in walking or moving his/her arms?	18/49 (36.7%)	5/42 (11.9%)
Does he/she have weakness and/or stiffness in the arms or legs?	14/49 (28.6%)	2/42 (4.8%)
At least one deficit	24/49 (48.9%)	7/42 (16.7%)

### Factors associated with MR

**Fig. 2:**
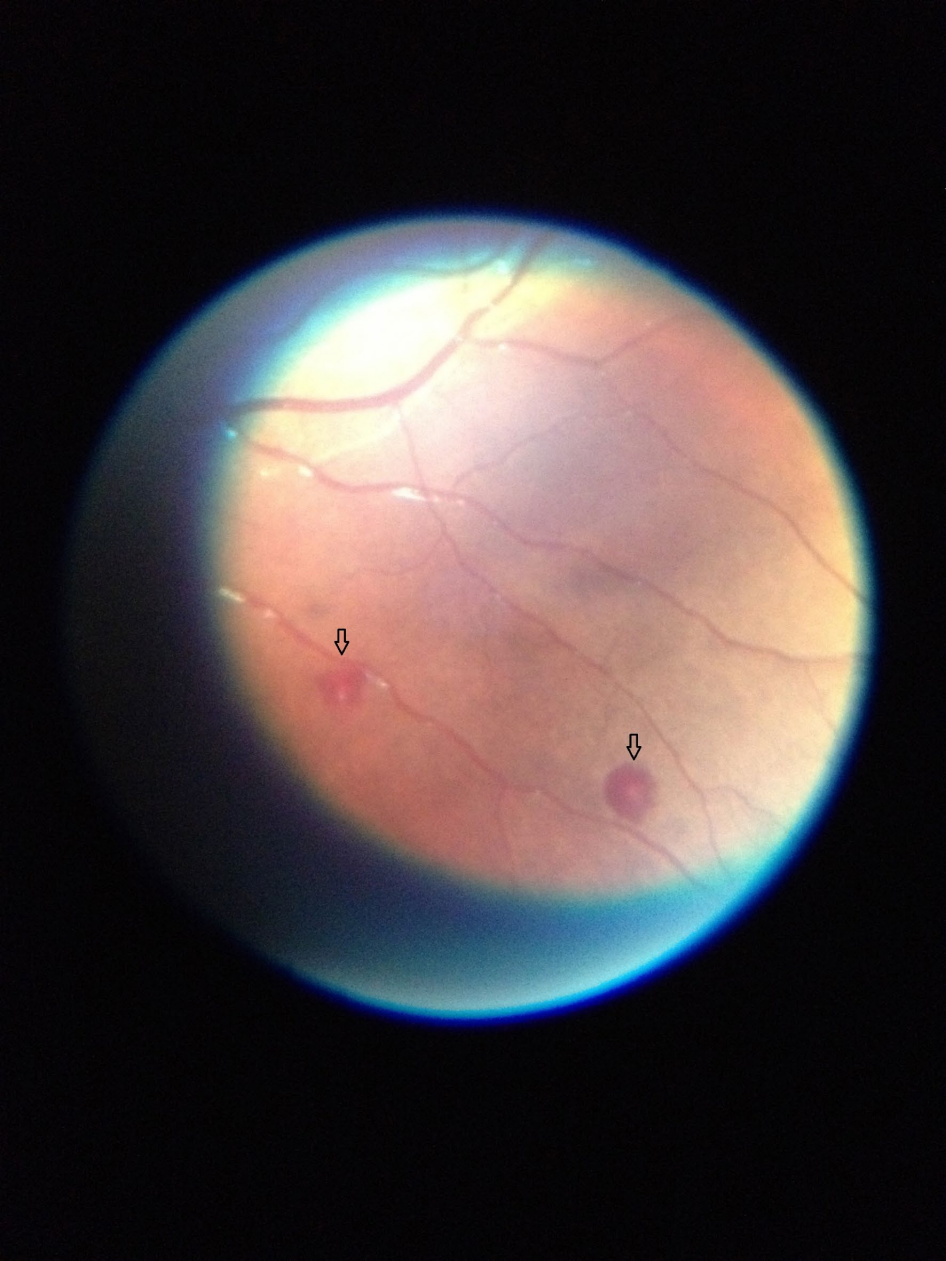
Fundus photograph of a paediatric patient showing white-centred retinal haemorrhages (see arrows).

Fundoscopy was carried out on D1 of hospitalization in 55 children with CM. Missing data in 15 children were due to death within 18 h after admission. Of these 55 children, 32 had at least one item defining MR. As shown in Fig. 2, the MR characteristics present in children were haemorrhages (30 children), whitening (4 children), and vascular changes (one child). Baseline characteristics associated with MR in univariate analysis are provided in Table 3 and Supplemental Table 1. The three factors significantly associated with an increased risk of MR were a traditional consultation before admission (81.8% of MR in children given traditional medicine vs. 42.4% of MR in children were not; *p* = 0.003), the absence of antiepileptic drug intake before admission (73.3% vs. 40.0%, *p* = 0.013), and severe anaemia at admission (77.3% vs. 44.1%, *p* = 0.014). In multivariate analysis, MR was associated with the admission site and a traditional consultation before admission (Table 4).

**Table 3:** Factors associated with MR, and neurocognitive deficits at discharge and at D21–D28.

Variables	With MR *n* = 32	*p*-value	NeuroCognitive deficits screener positive at discharge *n* = 24	*p*-value	NeuroCognitive deficits screener positive at D21–D28 *n* = 7	*p*-value
Age (*n* = 58)		0.376		0.921		0.390
4 years old or less	19/36 (52.8%)		16/33 (48.5%)		6/27 (22.2%)	
More than 4 years old	11/22 (65.0%)		8/16 (50.0%)		1/17 (6.7%)	
Gender (*n* = 56)		0.385		0.913		**0.099**
Female	21/34 (61.8%)		15/31 (48.4%)		2/25 (0.1%)	
Male	11/22 (50%)		9/18 (50.0%)		5/17 (29.4%)	
Admitting hospital (*n* = 56)		**0.054**		0.316		0.679
Calavi	13/29 (44.8%)		10/24 (41.7%)		2/18 (11.1%)	
CHUMEL	19/27 (70.3%)		14/25 (56.0%)		5/24 (20.8%)	
Medical consultation (*n* = 56)		0.446		1.000		**0.067**
Yes	29/49 (59.2%)		22/45 (48.9%)		5/39 (12.8%)	
No	3/7 (42.9%)		2/4 (50.0%)		2/3 (66.7%)	
Traditional consultation (*n* = 55)		0.003*		0.905		0.225
Yes	18/22 (81.8%)		10/20 (50.0%)		4/15 (26.7%)	
No	14/33 (42.4%)		14/29 (48.3%)		3/27 (11.1%)	
*Pre-admission treatment*						
Antibiotic (*n* = 56)		0.670		**0.083**		**0.075**
Yes	21/38 (55.3%)		19/33 (57.6%)		7/28 (25.0%)	
No	11/18 (61.1%)		5/16 (31.2%)		0/14 (0.0%)	
Anti-epileptic (*n* = 55)		0.013*		0.562		**0.093**
Yes	10/25 (40.0%)		12/22 (54.6%)		6/21 (28.6%)	
No	22/30 (73.3%)		12/26 (46.2%)		1/21 (4.8%)	
Onset of illness with coma (*n* = 56)		0.641		1.000		1.000
Yes	30/51 (58.8%)		22/45 (48.9%)		7/39 (17.9%)	
No	2/5 (40%)		2/4 (50.0%)		0/3 (0.0%)	
*Clinical and biological examinations*						
Blantyre’s score (*n* = 56)		0.573		0.496		1.000
2	26/44 (59.1%)		18/39 (46.2%)		6/33 (11.1%)	
0 or 1	6/12 (50%)		6/10 (60.0%)		1/9 (11.1%)	
Multiple convulsions (*n* = 55)		0.210		**0.083**		0.410
Yes	18/27 (66.8%)		9/24 (62.5%)		5/21 (23.8%)	
No	14/28 (50%)		9/24 (37.5%)		2/20 (10.0%)	
Respiratory distress (*n* = 56)		0.361		0.725		0.312
Yes	6/13 (46.1%)		5/9 (55.6%)		0/8 (0.0%)	
No	26/43 (60.5%)		19/40 (47.5%)		7/34 (20.6%)	
Jaundice^§^ (*n* = 55)		0.262		0.017*		**0.089**
Yes	15/23 (65.2%)		13/18 (72.2%)		5/16 (31.2%)	
No	16/32 (50.0%)		11/31 (36.7%)		2/25 (8.0%)	
Hypoglycaemia^**§**^ (*n* = 56)		0.338		0.675		**0.113**
Yes	7/15 (46.7%)		6/11 (54.6%)		3/8 (37.5%)	
No	25/41 (61.0%)		18/38 (47.4%)		4/34 (11.8%)	
Acidosis^§^ (*n* = 55)		0.660		1.000		1.000
Yes	13/21 (61.9%)		8/16 (50.0%)		2/14 (37.5%)	
No	19/34 (55.9%)		16/32 (50.0%)		5/27 (11.8%)	
Severe anaemia^§^ (*n* = 56)		0.014*		0.905		1.000
Yes	17/22 (77.3%)		10/20 (50.0%)		3/18 (16.7%)	
No	15/34 (44.1%)		14/29 (48.3%)		4/24 (16.7%)	
MR (*n* = 32)				0.005*		0.222
Yes			19/29 (25.0%)		6/26 (23.1%)	
No			5/20 (65.5%)		1/16 (6.3%)	

The table shows the percentages of children with retinopathy, neurocoginitive deficit at discharge and at D21-D28 for each variable subgroup (age over or under 4 years, gender M or F, etc.), and the significance of the comparison between the percentages obtained for each variable sub-group (p-value).

p-value < 0.20 are in bold. *p-value < 0.05.

^§^ Jaundice or elevated bilirubin > 50 µmol/L; Hypoglycaemia (glucose < 2.2 mmol/L); Acidosis (plasma bicarbonate < 15 mmol/L); Severe anaemia (haemoglobin < 50 g/L, haematocrit < 15%).

**Table 4:** Factors associated with MR and neurocognitive deficits at discharge in multivariate analyses.

Variables	With MR 32/56 (57.1%)	NeuroCognitive deficits at discharge 24/49 (48.9%)
	Adjusted OR (95% CI)	Adjusted OR (95% CI)
Admitting hospital		
Calavi	1	
CHUMEL	25.05 (2.39–262.99)*	
Traditional consultation		
Yes	25.32 (2.24–286.51)*	
No	1	
*Pre-admission treatment*		
Antibiotic		
Yes		2.97 (0.65–13.59)
No		1
Anti-epileptic		
Yes	0.36 (0.07–1.76)	
No	1	
*Clinical and biological examinations*		
*Multiple convulsions*		
Yes		2.03 (0.52–7.97)
No		1
Jaundice or elevated bilirubin > 50 µmol/L		
Yes		3.04 (0.73–12.67)
No		1
Severe anaemia (haemoglobin < 50 g/L, haematocrit < 15%)		
Yes	2.96 (0.65–13.55)	
No	1	
MR		
Yes		5.54 (1.30–23.54)*
No		1

p-value < 0.20 are in bold. *p-value < 0.05.

### Factors predictive of neurological sequelae at day of discharge and at D21–28 post-admission

In univariate analysis, jaundice and MR were significantly associated with neurological deficit at discharge (Table 3). By contrast, no baseline characteristic was significantly associated with MR at D21–D28 post-admission. In multivariate analysis, none of the exposure variables tested (antibiotic intake prior to admission, multiple convulsions, jaundice) showed a significant association with neurocognitive deficit at hospital discharge. On the other hand, neurological deficits at discharge were associated with MR (Table 4). Multivariate analyses at D21–D28 were not possible given the low number of observations.

### Association between immune factors and MR and neurocognitive deficit

**Fig. 3:**
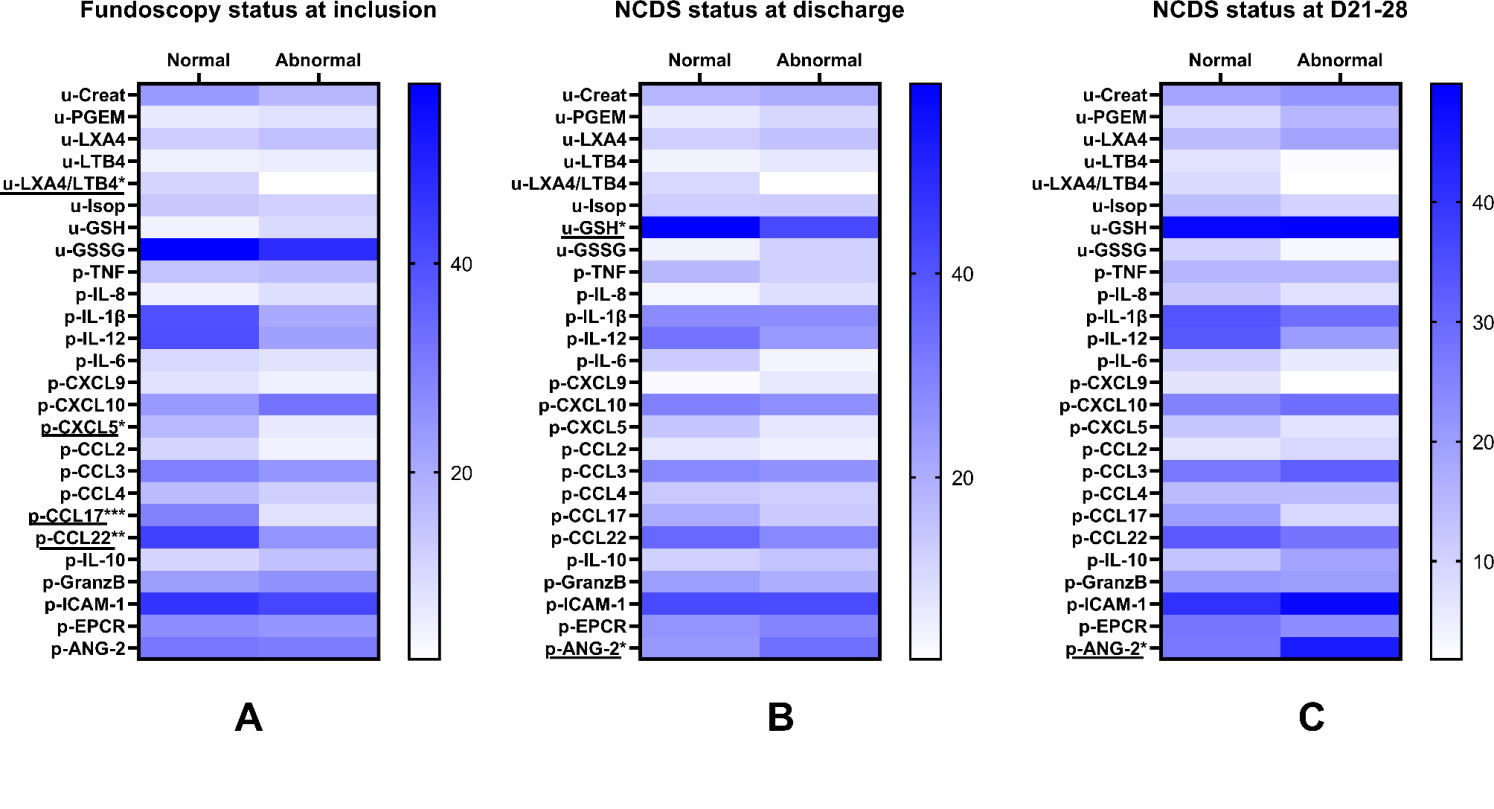
Association of urinary and plasma immune markers with fundoscopy status (**A**) and neurological deficits at discharge (**B**) and between 21 and 28 days post-admission (**C**). The heatmap presents the means of normalized values to the maximum value of each biomarker x 100. Detailed values are presented in Supplementary Tables 1 and 2. The Mann–Whitney U-test was used to test associations, * indicates *p* < 0.05.

Immune markers of inflammation, the oxidative response, and endothelium activation were measured in plasma and urine at inclusion to study their association with MR and neurologic impairment through univariate analysis. MR was associated with a lower urine LXA4/LTB4 ratio and plasma level of CXCL5, CCL17, and CCL22 (Fig. 3). Although both urine LXA4 and LTB4 levels were higher in the MR group, the lower ratio was due to higher levels of proinflammatory LTB4 (Table S1). Neurocognitive deficit at discharge was associated with lower urinary levels of glutathione (GSH), a known marker of the antioxidant state, and with higher plasma levels of angiopoietin-2, a marker of endothelium activation (Table S2). At D21–28 post-inclusion, only higher plasma levels of angiopoietin-2 were still associated with neurocognitive deficit.

## Discussion

The high proportion of MR at admission, along with the high proportion of neurological deficits at discharge, which substantially decreased by D21–28 post-admission, are the main findings of our study. Although no baseline characteristic was associated with a post-discharge deficit, MR at admission was associated with a neurocognitive deficit at discharge.

This study presents a cohort of severe comas in African children, the only one performed in West Africa. Although children were provided with emergency treatments including intravenous artesunate, a high fatality rate was observed (28.6%). This could be due to the fact that these children were in deep comas, possibly with multiorgan failure, and had no access to intensive care. No brain imaging was available and we may have missed, for example, cerebral tumours or bleeding as a cause of coma, as nonmalarial causes may coexist with parasitaemia^27^. However, we are confident that the diagnoses of CM were exhaustive and reliable, due to the high quality of laboratory testing and the use of a panel of experts to ensure the best classification of the patients^2^.

Some limitations of this study should be mentioned. Because most of children who died had missing fundoscopic data, the proportion of children with MR may have been biased and potentially underestimated. Indeed, it is likely that children who died were at higher risk of MR. Data on clinical history, based on interviews with parents/guardians at admission and a health booklet review may not have been exhaustive. Neurodevelopmental deficits were assessed through a questionnaire derived from the TQ. This screener was associated with the Mullen Scales of Early Learning at 1 year of age^28^ and the Kaufman Assessment Battery for Children – Second edition – at 6 years of age in a Beninese cohort study (R. Zoumenou, personal communication). However, in our study, we included only a subsample of the TQ questions and referred to the child’s state before CM. In addition, although the TQ is efficacious for screening for neurodevelopmental deficits, it reflects caregiver perception and is not an objective test administered by a trained psychologist. Therefore, declared neurological deficits may be affected by caregiver perception, especially in the context of CM. Our study used the TQ, a screener for neurodevelopmental deficits, but a robust measure of neurocognitive deficits would have been useful, like the Kaufman Assessment Battery for Children. A study in Uganda validated the TQ and reported a positive rate of 27%^29^. The TQ screen is considered positive if any 1 of 10 TQ questions is positive. The rate of neurological deficits was 48.9% at discharge and 16.7% at 21–28 days post-admission. Given that we included only 8 of the 10 TQ questions, the percentage of neurological deficits in our study may have been slightly higher if we had included all questions. The questions related to seizures and learning were not included. Therefore, our results seem consistent with the study in Uganda. Finally, our study may have lacked power to extensively identify the factors associated with death and neurological outcomes. For several variables, confidence intervals were large and should be interpreted with caution. Seventy-two participants would have been needed to be able to detect an association between MR and neurological deficits at D21–28 with an Odds Ratio of 5 and a power of 80%.

Depth of coma and some established biological criteria for severe malaria at admission were associated with a fatal outcome in this cohort. We have no explanation as to why boys died more frequently, however, male sex has been associated with a greater risk for new neurodisability in MR-positive paediatric CM survivors^4^. Death occurred shortly after admission in most cases, before any emergency antimalarial prescription could have been effective and may explain why mortality remains high in the artesunate era.

MR was first described about 30 years ago^30^ and fundoscopy is rarely a part of CM management in routine practice^11^. We decided not to include MR in the definition of CM, in line with the current WHO criteria for CM^31^. While performing a fundoscopy within 24 h after admission was planned for each admitted child, a substantial proportion of children could not be assessed for MR because they died very early after admission and before the research clinician was available at the study site. This illustrates the difficulties of efficiently implementing fundoscopy in the field to help manage CM. Most children had an MR and the most common feature was haemorrhage. An autopsy study in Malawi found that severity of haemorrhage in the retina correlates with that in the brain^32^. Our data enabled us to explore the association between characteristics observed at admission and MR in patients with CM. A consultation with a traditional practitioner before admission was associated with MR. However, there was no association between the duration from the onset of symptoms to hospital admission and consultations with a traditional practitioner, which could explain this association. In addition, MR was independent of any delay between the first symptoms and admission. This result could therefore suggest either that traditional consultations contributed to the development of retinopathy, or that retinopathy itself influences the need for traditional consultations. Interestingly, not taking antiepileptic drugs was associated with MR. As the onset of multiple convulsions (treated with antiepileptic drugs) was not associated with MR, this result may reflect the difficulty of accessing conventional care, which is in line with traditional medicine consultations and favours MR. MR was also associated with severe anaemia, an established severe malaria criterion, in univariate analysis. Lewallen et al.. found a significantly lower haematocrit level in children with retinal haemorrhage^33^. Furthermore, Chillaverde et al.. showed that children with MR have a significantly lower haemoglobin level at admission than children without it^34^. Overall, our results add to the already described association between MR and established criteria for severe malaria.

Our findings corroborate previous reports^9,35^, showing that disabilities are much less prevalent a few weeks after discharge than at discharge. Most reported issues at admission were related to motor function. These are also the functions that seemed to have improved by D21–28. Therefore, the acute phase of CM had an important impact on motor function. Future studies should include neuropsychological tests administered by trained assessors to be able to capture detailed cognitive abilities that may be affected by CM. Because epidemiological studies performed in malaria-endemic areas have found some neurological disabilities in children with no history of severe malaria^29,36^, all of the neurological deficits detected in our study may not be attributable to malaria. Some of them likely pre-existed before CM.

Few studies have explored the interactions between MR and neurological outcomes after CM. In studies in Malawi that carried out 18 months of follow-up for neurological assessment after CM, a high proportion of deficits (epilepsy, other neurobehavioral sequelae) was found in both children with and without MR^4,16^ but there was no trend toward a higher risk of obvious neurological sequelae in those with MR (132 children) compared to those without it (35 children)^16^. By contrast, another study also in Malawi, where fundoscopy was performed in 49 children and severity of MR was scored, reported a relationship between MR severity and persistent neurocognitive problems^8^, suggesting a link between MR at admission and neurological outcomes. While jaundice at admission, a characteristic associated with death over the whole cohort, was associated with neurological deficits at discharge in univariate analysis, MR was the only baseline characteristic associated with neurological deficits at discharge in both univariate and multivariate analyses. Thus, our data add to previous evidence of an association between MR and neurological outcomes, assessed at discharge in this population, suggesting that MR may be a marker of brain injury^32^.

We also focused on immune biomarkers of both neurological sequelae after CM and MR, measured in plasma and urine. Only a few studies have evaluated links between immunologic markers and neurologic deficits after CM. John et al.. reported a correlation between elevated levels of TNF in cerebrospinal fluid (CSF), but not in serum, and increased risk of neurologic deficit 3 months after admission in children with CM^37^. This association was confirmed by two studies reporting neurologic deficits at discharge and 3 months after CM (in the first study^38^) and 2 years after a severe malaria episode, including CM (in the second study^39^). Plasma and serum proinflammatory cytokines have also been studied in relation to neurologic deficits in many clinical pathologies in children and newborns. Elevated serum levels of proinflammatory cytokines (IL-1β, IL-6, TNF) and markers of central nervous system impairment (enolase, S-100 protein) have been described in hypoxic–ischemic conditions in newborns at high risk for neurologic sequelae^40^. Elevated plasma IL-6 levels are also associated with neuromotor abnormalities in children 6 months after open-heart surgery, an intervention known to cause frequent long-term neurodevelopmental sequelae^41^. In our study, no such relation was found between proinflammatory cytokines (TNF, IL-1β, IL-8, IL-12, IL-6) and neurological deficit studied at discharge or 28 days after inclusion. This could be due to our short follow-up of less than 1 month. However, Shabani et al.. observed a correlation between CSF levels of TNF and neurological deficits at discharge in CM children. Cytokines in the CSF may better reflect neuroinflammation than those in the plasma, although CSF sampling is not easy, particularly in CM children, and difficult to implement in routine clinical practice. Interestingly, we found that elevated plasma levels of angiopoietin-2, a marker of endothelial activation, were associated with neurologic deficit in children at discharge and at D21–28. Angiopoietin-2 was previously found to be associated with cognitive injury over 2 years after a severe malaria episode in children^42^, and it is widely accepted as a biomarker of malaria severity^19,43,44^. In our study, at discharge, but not at D21–28, urine levels of reduced glutathione (GSH) were lower in children with neurologic deficit. To the best of our knowledge, this is the first report of such an association after severe malaria. This result highlights the importance of the antioxidant response in resolving neuroinflammation during CM. A study carried out in children presenting with acute lymphoblastic leukaemia, a disease associated with a high risk for cognitive impairment, also reported that higher initial GSH levels, measured in CSF, were associated with better cognition over 1 year of follow-up^45^, underlining the weight of the antioxidant response in the neurologic state.

In addition, plasma and serum markers are inconsistently related to MR. Villaverde et al.. reported similar plasma levels of proinflammatory cytokines (TNF, IFNϒ, IL-1β, IL-10) and oxidative stress markers in both MR-positive and MR-negative children with CM^46^. In contrast, in nonmalarial retinopathy such as retinopathy of prematurity and diabetic retinopathy, the inflammatory response is consistently associated with retinopathy^47–49^. In a murine model of diabetic retinopathy, M2 polarization of macrophages via IL-10 was even found to reduce retinopathy^49^. In line with this, we found that MR was associated with a lower anti-inflammatory response, which resulted in a lower LXA4/LTB4 ratio, CCL17 and CCL22 levels (two chemokines implicated in Th2 responses), and regulatory T cell migration^21^. The CXCL5 level, which is implicated in neutrophil trafficking, was also lower in MR-positive patients. This result is surprising, because the neutrophil response participates in the inflammatory response to limit infection but also contributes to neuroinflammation and tissue damage and is a therapeutic target in ocular diseases^50^. However, a study conducted in Malawi demonstrated impaired neutrophil chemotaxis in MR-positive versus MR-negative patients, despite higher levels of inflammatory cytokines and neutrophil activation^51^. The authors attributed this failure to the presence of free haem in plasma, known to decrease neutrophil migratory capacity via haem-oxygenase activity^52–54^. Such a paradoxical result may be due to the specificity of malarial infection.

## Conclusion

In a population of children with CM admitted in tertiary care hospitals, a high fatality rate was found. MR at admission was associated with the use of traditional medicine and with the study site. These factors may translate into difficulties accessing conventional care. Neurological deficits were strikingly more prevalent at discharge than a few weeks later. MR at admission was predictive of neurological deficit at discharge, while endothelium activation was associated with neurological deficit both at discharge and at D21–28. The NeuroCM study mainly explored inflammation in comatose vs. non-comatose children infected with malaria and therefore included a large battery of immunological indicators^2^. This allowed the study of associations between these indicators and health outcomes, MR, and neurological deficit. Further studies on neurological outcomes of CM should be adequately powered to provide more robust analysis of determinants of disabilities observed early after discharge and also to explore cognitive abilities that may be affected by CM.

## Methods

### Study design and participants

This prospective study was conducted at two university hospitals from March 1 to November 30, 2018: the Centre Hospitalier Universitaire de Zone d’Abomey Calavi/Sô-Ava (CHUZ-AS) and Centre Hospitalier Universitaire de la Mère et de l’Enfant Lagune (CHU-MEL) in Cotonou, Benin. A clinical research physician in each hospital was specifically trained to manage the NeuroCM study. Patient management and the investigations performed to assess the aetiology of coma are detailed elsewhere^2^.

The inclusion criteria were children aged 24–71 months with nontraumatic coma, evidence of *P*. *falciparum*infection in blood smears and/or PCR, a negative result in human immunodeficiency virus (HIV) rapid RDT, and a Blantyre Coma Score (BCS) ≤ 2. The exclusion criteria were the absence of parental consent, any coinfection (as detected via blood and cerebrospinal fluid culture, serology tests, and multiplex PCR), pre-existent neurological disease, and traumatic or toxic or nontraumatic nonmalarial coma (see details in reference^2^).

### Ethics review and approval

Ethics approval for the NeuroCM study was obtained from the Comité National d’Ethique pour la Recherche en Santé of Benin (n°67/MS/DC/SGM/DRFMT/CNERS/SA; 10/17/2017). The study was approved by the Comité consultatif de déontologie et d’éthique of Institut de Recherche pour le Développement (IRD; 10/24/2017). Written informed consent was obtained from the parents or guardians of all included children. All study procedures were performed in accordance with the institutional policies, guidelines and regulations pertaining to research involving human subjects.

### Fundoscopy

Within 24 h after admission, direct ophthalmoscopy was performed by a trained clinical research physician, with an EyePax 1.0 Dioptrix® ophthalmoscope, to determine MR status. Imaging of the exam was made possible with an adaptor on a mobile phone. Images were interpreted retrospectively and independently by two experienced ophthalmologists; in cases of disagreement, the expertise of a third ophthalmologist was required. MR was defined as the presence of retinal whitening, haemorrhage, or vascular changes with or without papilledema^11^.

### Neurocognitive deficits screener

A questionnaire was administered by a research clinician to the caregiver at discharge and between days 21 and 28 post-discharge (D21–28), using a questionnaire derived from the Ten Question questionnaire (TQ), a screening tool already used in Benin^55^. The questionnaire was administered to the child’s parents/guardians to gather information on their perception of neurodevelopmental deficits compared to before CM. The questionnaire included 8 of the 10 questions of the TQ. It included the following neurodevelopmental dimensions: standing, walking, sitting, vision, hearing, receptive language, tonus, epileptic crisis, expressive language, and mental retardation. A neurological deficit was considered as present when at least one difficulty among these was present. For the assessments on days 21–28, the parents/guardians were invited to come back to the hospital but the assessment was performed at home if necessary.

### Plasma biomarkers

Plasma levels of 17 biomarkers were measured as described previously^19^, using Luminex technology and the Human Premixed Multi-Analyte Kit (LXSAHM-17, R&D Systems, Lille, France). For plasma soluble EPCR, concentration was determined via ELISA according to the manufacturer’s recommendations (DuoSet, R&D Systems).

### Statistical analysis

In descriptive analysis, qualitative variables are expressed as numbers (%), and quantitative variables are given as mean ± standard deviation (SD) when they followed a normal distribution and as median (interquartile range) when they did not. In comparative analysis, comparison of proportions was performed using Pearson’s chi-square test or Fisher’s exact test. The Mann–Whitney U-test was used to compare quantitative variables without a normal distribution. The McNemar test was used to compare the results of neurological assessments performed at discharge and at D21–28 post-discharge (neurocognitive deficits screener) to determine whether the proportions of the paired data obtained were statistically different. To identify predictive factors of health outcomes (MR and neurological deficits), a logistic regression model was performed. Variables associated with a p-value < 0.20 in univariate analyses were included in the model. The threshold of significance was set to 5% (*p* < 0.05). SAS 9.4 software was used.

## Electronic supplementary material

Below is the link to the electronic supplementary material.

Supplementary Material 1

## Data Availability

The datasets used and analysed during the current study are available from the corresponding author on reasonable request.
