# Supplementary material for: Determinants of retinopathy and short-term neurological outcomes after cerebral malaria
Source: Sci Rep. 2025 Apr 19;15:13610. doi: 10.1038/s41598-025-97468-4 (PMC12009428; doi:10.1038/s41598-025-97468-4)
Supplement: Supplementary file 1 — Supplementary Material 1 [file 41598_2025_97468_MOESM1_ESM.docx]

**Additional files**

**Table S1** Association between urine and plasma immune mediators at inclusion and retinopathy

| **Urine/plasma** | **Biomarker** | **Normal retino.**  **n = 23** | **Abnormal retino.**  **n = 32** | ***P*** |
| --- | --- | --- | --- | --- |
| urine | Creatinine | 0.6 [0.2 - 1.9] | 0.5 [0.1 - 1.2] | ns |
| urine | PGEM | 0.4 [0.1 - 0.9] | 0.5 [0.08 - 1.6] | ns |
| urine | LXA4 | 0.6 [0.4 - 1.6] | 1.8 [0.5 - 3.6] | ns |
| urine | LTB4 | 0.5 [0.3 - 3.9] | 3.1 [1.2 - 8.0] | ns |
| urine | **LXA4/LTB4** | 0.8 [0.3 - 1.5] | 0.4 [0.3 - 0.7] | **0.009** |
| urine | Isoprostane | 4.6 [2.6 - 9.2] | 5.0 [3.0 - 19.0] | ns |
| urine | GSH | 1.3 [0.7 - 1.6] | 0.9 [0.5 - 1.2] | ns |
| urine | GSSG | 0.001 [0.001 - 0.006] | 0.004 [0.001 - 0.01] | ns |
| plasma | TNF | 39.2 [9.1 - 78.6] | 31.7 [18.1 - 58.8] | ns |
| plasma | IL-8 | 45.4 [20.4 - 69.2] | 24.0 [9.9 - 69.8] | ns |
| plasma | IL-1β | 19.3 [9.3 - 41.4] | 9.3 [5.9 - 16.0] | 0.06 |
| plasma | IL-12 | 115.7 [73.6 - 170] | 56.6 [56.6 - 65.1] | ns |
| plasma | IL-6 | 93.2 [24.2 - 218] | 54.8 [30.0 - 152.9] | ns |
| plasma | CXCL9 | 1 340 [716 – 2 539] | 1 247 [716 - 2 936] | ns |
| plasma | CXCL10 | 267 [109 - 467] | 321 [192 - 576] | ns |
| plasma | **CXCL5** | 540 [313 – 1 860] | 313 [210 - 756] | **0.04** |
| plasma | CCL2 | 154 [74.7 - 383] | 130 [45.2 - 339] | ns |
| plasma | CCL3 | 327 [257 - 327] | 257 [161 -327] | ns |
| plasma | CCL4 | 306 [149 - 617] | 236 [173 - 429] | ns |
| plasma | **CCL17** | 153 [114 - 388] | 67.2 [67.2 - 114] | **<0.0001** |
| plasma | **CCL22** | 515 [367 - 783] | 384 [203 - 469] | **0.004** |
| plasma | IL-10 | 350 [65 - 762] | 380 [154 - 861] | ns |
| plasma | GranzB | 415 [121 - 1 338] | 495 [305 -1 069] | ns |
| plasma | ICAM-1 | 1.3 x10^6^ [0.8x10^6^ -1.8x10^6^] | 1.0x10^6^ [0.7x10^6^ - 1.5x10^6^] | ns |
| plasma | EPCR | 9.1 [5.0 - 24.5] | 13.1 [6.9 - 19.3] | ns |
| plasma | ANG-2 | 16 507 [11 039 - 20 248] | 16 583 [11 456 - 26 997] | ns |

Values are geometric means [25^th^ – 75^th^ percentile] in ng/mL

**Table S2** Association between plasma cytokine levels and other soluble mediators at inclusion and neurocognitive deficits screener (NCDS) at discharge and at D21-28 post-inclusion

|  | **At discharge** | | | | | **At D21-28 post-inclusion** | | | | |
| --- | --- | --- | --- | --- | --- | --- | --- | --- | --- | --- |
| **ng/mL** | **n** | **Normal NCDS** | **n** | **Abnormal NCDS** | ***P*** | **n** | **Normal NCDS** | **n** | **Abnormal NCDS** | ***P*** |
| Creatinine | 25 | 0.6 [0.2 - 1.3] | 23 | 0.3 [0.1 - 1.3] | ns | 35 | 0.5 [0.1 - 1.2] | 7 | 0.2 [0.1 - 1.3] | ns |
| PGEM | 25 | 0.5 [0.08 - 1.1] | 23 | 0.9 [0.1 - 1.6] | ns | 35 | 0.5 [0.1 - 1.5] | 7 | 1.5 [0.2 - 2.3] | ns |
| LXA4 | 25 | 0.9 [0.4 - 3.1] | 23 | 1.7 [0.5 - 3.5] | ns | 35 | 1.3 [0.5 - 3.3] | 7 | 2.2 [0.2 - 6.4] | ns |
| LTB4 | 25 | 1.5 [0.4 - 4.5] | 23 | 2.9 [0.6 - 11.9] | ns | 35 | 1.8 [0.5 - 6.9] | 7 | 1.3 [0.5 - 13.5] | ns |
| LXA4/LTB4 | 25 | 0.6 [0.3 - 1.6] | 23 | 0.5 [0.3 - 0.7] | ns | 35 | 0.4 [0.3 - 1.0] | 7 | 0.7 [0.3 - 0.8] | ns |
| Isoprostane | 25 | 5.2 [3.1 - 16.2] | 23 | 4.7 [2.4 - 23.2] | ns | 35 | 5.3 [3.2 - 19.0] | 7 | 5.8 [1.9 - 17.8] | ns |
| **GSH** | 25 | 1.3 [0.8 - 1.6] | 23 | 0.8 [0.4 - 1.2] | **0.04** | 35 | 1.0 [0.5 - 1.5] | 7 | 0.9 [0.8 - 1.2] | ns |
| GSSG | 25 | 0.001 [0.001 -0.05] | 23 | 0.006 [0.001 -0.01] | ns | 35 | 0.003 [0.001 -0.01] | 7 | 0.006 [0.001 -0.008] | ns |
| TNF | 24 | 45.2 [12.6 - 64.9] | 24 | 30.2 [17.7 - 56.5] | ns | 34 | 34.7 [15.6 - 64.9] | 7 | 41.0 [14.1 - 63.8] | ns |
| IL-8 | 24 | 26.0 [16.7 - 79.9] | 24 | 28.1 [10.6 - 59.1] | ns | 34 | 28.6 [17.3 - 77.2] | 7 | 19.3 [13.7 - 51.0] | ns |
| IL-1β | 13 | 9.3 [5.9 - 27.3] | 14 | 14.3 [5.9 - 19.3] | ns | 18 | 14.3 [5.9 - 22.5] | 6 | 7.6 [5.9 - 16.0] | ns |
| IL-12 | 9 | 116 [56.6 - 123] | 7 | 56.6 [56.6 - 90.7] | ns | 12 | 103 [56.6 - 123] | 2 | 73.6 [56.6 - 90.7] | ns |
| IL-6 | 24 | 90.3 [27.5 - 315] | 23 | 54.8 [25.2 - 162] | ns | 34 | 55.2 [25.0 - 293] | 6 | 65.7 [25.9 - 117] | ns |
| CXCL9 | 19 | 1 672 [716 - 3 167] | 23 | 1 155 [716 - 3 087] | ns | 29 | 1 672 [716 - 3245] | 7 | 716 [716 - 1654] | ns |
| CXCL10 | 15 | 184 [89.4 - 654] | 16 | 357 [192 - 585] | ns | 21 | 249 [108 - 542] | 6 | 408 [321 - 479] | ns |
| CXCL5 | 24 | 614 [281 - 1382] | 24 | 423 [246 - 794] | ns | 34 | 423 [270 - 1205] | 7 | 523 [320 - 831] | ns |
| CCL2 | 22 | 142 [58.5 - 383] | 23 | 134 [50.9 - 510] | ns | 32 | 95.6 [26.2 - 491] | 7 | 154 [54.1 - 627] | ns |
| CCL3 | 19 | 257 [185 - 327] | 20 | 257 [161 - 380] | ns | 28 | 257 [161 - 327] | 5 | 327 [257 - 476] | ns |
| CCL4 | 23 | 273 [161 - 538] | 20 | 286 [196 - 617] | ns | 32 | 273 [173 - 617] | 6 | 320 [149 - 637] | ns |
| CCL17 | 20 | 114 [67.2 - 204] | 17 | 67.2 [67.2 - 114] | 0.08 | 25 | 114 [67.2 - 196] | 7 | 67.2 [67.2 - 114] | ns |
| CCL22 | 24 | 474 [305 - 563] | 23 | 395 [261 - 505] | ns | 33 | 410 [255 - 554] | 7 | 416 [291 - 523] | ns |
| IL-10 | 24 | 511 [131 - 682] | 24 | 358 [139 - 1 780] | ns | 34 | 446 [137 - 1 086] | 7 | 351 [110 - 703] | ns |
| GranzB | 22 | 481 [101 - 1 332] | 21 | 493 [279 - 971] | ns | 30 | 443 [133 - 1 069] | 7 | 493 [243 - 1 284] | ns |
| ICAM-1 | 22 | 1.3x10^6^ [0.7x10^6^ -1.6x10^6^] | 22 | 1.1x10^6^ [0.7x10^6^ -1.6x10^6^] | ns | 31 | 1.2x10^6^ [0.7x10^6^ -1.6x10^6^] | 6 | 1.1x10^6^ [0.9x10^6^ -1.8x10^6^] | ns |
| EPCR | 24 | 7.3 [0.06 – 27.5] | 24 | 14.5 [6.9 - 25.4] | ns | 34 | 10.9 [5.6 – 26.4] | 7 | 10.7 [1.0 - 22.0] | ns |
| **ANG-2** | 24 | 14 648 [9 876 - 18 321] | 24 | 16 583 [12 687 - 27 268] | **0.05** | 34 | 14 468 [10 592 - 20 323] | 7 | 23 606 [16 352 -35 722] | **0.05** |

Values are geometric means [25^th^ – 75^th^ percentile]
